# Effects of synthetized porcine follicle-stimulating hormone and synthetized human chorionic gonadotropin on reproductive efficiency in pigs

**DOI:** 10.1186/s40813-025-00476-z

**Published:** 2025-12-29

**Authors:** Jiahao Li, Wenjun Zhu, Chengnuo Hu, Wenchao Wu, Xuelong Liu, Zheng Huang, Li Li, Haoshu Luo, Hengxi Wei, Shouquan Zhang

**Affiliations:** 1 State Key Laboratory of Swine and Poultry Breeding Industry, National Engineering Research Center for Breeding Swine Industry, Guangdong Provincial Key Lab of Agro-animal Genomics and Molecular Breeding, College of Animal Science of South China Agricultural University, Guangzhou, 510600 China; 2 College of Biology and Food, Shangqiu Normal University, Shangqiu, 476000 China; 3 Beijing VJTBio Co., LTD, Beijing, 100085 China

**Keywords:** Synthetized human chorionic gonadotropin, Synthetized pig follicle-stimulating hormone, Promote estrus, Inducing puberty, Reproductive efficiency

## Abstract

This study aimed to evaluate the application of synthetized porcine follicle-stimulating hormone (spFSH) in combination with synthetized human chorionic gonadotropin (shCG) in inducing puberty in gilts, promoting estrus and ovulation in multiparous sows, and improving reproductive performance. In gilts, treatment with 30 µg spFSH + 150 IU shCG advanced the onset of puberty to 160 ~ 175 days of age in 76.62% of gilts, with a significantly lower incidence of ovarian cysts compared to PMSG and PG600. Hormonal profiling demonstrated that the applied protocol successfully induced endocrine maturation in gilts to levels consistent with those characteristic of natural puberty. In multiparous sows, the estrus-inducing effect of 30 µg spFSH + 150 IU shCG was comparable to PMSG and PG600 (400 IU PMSG and 200 IU hCG), while the incidence of ovarian cysts was significantly lower than in the PG600 group. This treatment significantly increased total piglets born per litter and reduced the number of days each piglet requires to be raised by a sow. When 300 IU shCG was used for ovulation induction, the farrowing rate significantly increased, and the number of days each piglet requires to be raised by a sow were further reduced. Hormonal analysis revealed that this treatment achieved physiological regulation of progesterone (P_4_) levels, with endocrine changes better aligned with sow reproductive physiology. This study systematically evaluated the application value of spFSH in combination with shCG at different doses in the reproductive management of gilts and sows. The optimized combination significantly improved estrus expression and farrowing rates, reduced ovarian cyst incidence, improving economic efficiency, and enhanced reproductive efficiency.

**Supplementary Information:**

The online version contains supplementary material available at 10.1186/s40813-025-00476-z.

## Background

Fixed-time artificial insemination (FTAI) is an effective assisted reproductive technology that does not require estrus detection and can help solve the problem of low breeding efficiency in pig farms. Among these, the use of reproductive hormones is a key factor in achieving successful batch management [1]. Although the combination of eCG and hCG is widely recognized for its effectiveness, their practical application in sow batch production is often limited by issues related to source, purification process, and inconsistent efficacy [2, 3]. Therefore, there is need in the industry to develop novel hormonal protocols that are more efficient, safe, stable, and cost-effective.

Under natural conditions, early puberty in sows usually reflects their physiological maturity and sufficient nutritional reserves, laying the foundation for full development of their reproductive potential; although early puberty is not necessarily caused directly by hormones, exogenous reproductive hormones can be used to promote its early occurrence [4]. Studies have demonstrated a significant correlation between the age at first estrus and reproductive performance in gilts [5]. Earlier attainment of puberty is associated with faster improvements in productivity and reduced non-productive days. Among traditional methods, PG600 has shown favorable effects in inducing estrus in anestrous gilts; however, its high cost and sensitivity to follicular developmental stages result in inconsistent efficacy, which limits its broader application in animal reproduction [6, 7]. In multiparous sows, pregnant mare serum gonadotropin (PMSG) and follicle-stimulating hormone (FSH) are commonly used to promote estrus, but both present substantial limitations. Commercial PMSG, derived from serum extracts, is constrained by limited supply, difficulties in purification, and potential risks of disease transmission [8]. Additionally, PMSG possesses fixed and potent FSH- and LH-like activities in non-equine species, which, upon long-term use, may cause endocrine imbalance, reduce estrus induction efficacy, increase the incidence of ovarian cysts, and lower embryo survival and pregnancy rates [9–13]. Traditional FSH, primarily extracted from animal pituitary glands, also suffers from limitations such as immunogenicity, low biological activity, poor purity, impurities, and a short half-life of 2 ~ 4 h [14, 15]. In comparison, synthetized porcine FSH (spFSH - also denominated as recombinant porcine FSH) produced via Chinese hamster ovary (CHO) cells offers improved pharmacokinetics, including prolonged half-life and more stable in vivo concentrations, which can enhance estrus induction and follicular development [16–18]. These advantages suggest spFSH has the potential to replace conventional hormones, though its practical application in sow production still requires further validation [18, 19].

Inappropriate timing of insemination is one of the major factors affecting sow reproductive performance. When fertilization ability is guaranteed, the oocyte can survive for about 12 h after ovulation, and the sperm can survive in the fallopian tube for about 24–48 h after insemination [20, 21]. This temporal mismatch is a key contributor to reduced conception rates [22]. Therefore, insemination shortly before but close to the time of ovulation is essential for achieving high fertility. At present, FTAI mainly relies on the use of ovulation-inducing hormones. Gonadotropin-releasing hormone (GnRH) analogs, such as buserelin and gonadorelin, induce ovulation indirectly by stimulating pituitary release of FSH and LH; however, their efficacy depends on the pituitary LH reserve and is therefore unstable [23, 24]. FSH induces luteinizing hormone/choriogonadotropin receptors (LHCGR) expression in granulosa cells during late follicular development, thereby enhancing follicular sensitivity to the LH surge and triggering the transition from FSH dependence to LH dependence leading to ovulation [25]. Although ovulation is not entirely dependent on the LH surge, a weakened LH peak may compromise the quality of follicular luteinization [26]. In contrast, hCG acts independently of the pituitary by directly activating ovarian LHCGR, thereby mimicking the endogenous LH surge and effectively synchronizing ovulation [27, 28]. However, traditional urine-derived hCG (uhCG) has the disadvantages of complex production process, high cost and large batch-to-batch variability. Its degradation products may interfere with oocyte maturation and reduce gonadotropin activity, thereby shortening the in vivo effectiveness [3, 29–31]. Synthetized hCG (shCG - also denominated as recombinant hCG), produced via CHO cell expression systems, offers higher purity and stability, and even at low doses can effectively promote follicular maturation with physiological effects comparable to high-dose uhCG while improving local tolerability [32–34].

In summary, gonadotropic hormones have been used in the reproductive management of sows to improve the reproductive efficiency and productive performance of both gilts and multiparous sows [35]. However, current hormonal protocols may impair uterine receptivity and reduce embryo implantation rates, ultimately affecting overall reproductive outcomes [10]. In this study, spFSH and shCG, both produced via CHO cell expression systems, were selected to balance biological activity, safety, and cost-effectiveness in optimizing sow reproductive management. Given that different dosage combinations may elicit distinct biological responses, this study systematically evaluated and identified the optimal combination strategy. The aim was to achieve maximal effectiveness in inducing puberty in gilts, promoting estrus and ovulation in multiparous sows, and improving reproductive efficiency, thereby providing a theoretical basis for the application of these synthetized hormones in commercial pig production.

## Materials and methods

### Experimental design

All procedures followed the guidelines for the ethical treatment of animals and were approved by the Animal Ethics Committee of the South China Agricultural University (approval number SCAU#0025).

#### Experiment on inducing puberty in gilts

The experiment was conducted from September to December 2023 at a large-scale conventional pig farm located in central Guangdong, China. During the study period, the average daily temperature ranged from 20.0 °C to 30.0 °C, with a relative humidity of 70% to 80%. The study subjects were sows in good health and of similar body condition, while sick or abnormal sows were excluded. A total of 647 hybrid sows (Landrace × Large White) with similar body weight (approximately 100–120 kg) and backfat thickness (14–20 mm) were purchased from Guangzhou Pucheng Biotechnology Co., Ltd. (Guangdong, China) at 100 days of age. All sows were housed in a conventional semi-intensive system, with 10 sows per pen, and no mating or ovulation induction was performed before the experiment. Starting from approximately 140 days of age, gilts are subjected to boar pheromone induction for approximately 20 days and estrus behavior is observed simultaneously. When the sows reach 160–175 days of age and the group estrus rate exceeds 30%, those that have not yet shown estrus will be transferred to individual pens for breeding. After the transfer, sows with obvious follicular development or clear corpus luteum were excluded by ultrasound examination, and those with obvious swelling of the vulva were excluded by visual inspection. Double-blind experimental design was used during the experiment.

The trial was conducted in the following sequential steps: (1) Optimization of spFSH dose: A total of 144 gilts were used in this part, 30 µg spFSH was finally determined to be the optimal dose (see Supplementary Material A for details) (2). Optimization of shCG dose: A total of 210 gilts were used in this part, 150 IU shCG was finally determined to be the optimal dose (see Supplementary Material B for details) (3). Comparison of hormone regimens for inducing puberty: In the third stage, 293 prepubertal gilts that had not yet expressed estrus were randomly assigned into four groups: control (77 prepubertal gilts; 5 ml physiological saline); PMSG group (800 IU/gilt; Ningbo Second Hormone Factory, Zhejiang, China) (62 prepubertal gilts); spFSH + shCG group (administered at the optimal combination dose identified from previous trials; Beijing VJTBio Co., LTD, Beijing, China) (77 prepubertal gilts); and PG600 group [(PMSG 400 IU + hCG 200 IU)/5 ml/gilt; Intervet International Ltd., Boxmeer, Netherlands] (77 prepubertal gilts). Daily estrus monitoring continued as described above to compare the effectiveness of different hormonal protocols in inducing puberty. For serum hormone assays(E_2_、FSH、LH、P_4_), three gilts were selected from each group (12 gilts in total), and blood samples were collected at 13 time points: 8 h before treatment and 24, 32, 48, 56, 72, 80, 96, 104, 120, 128, 144, and 168 h post-treatment (with the time of administration designated as 0 h). According to statistical power analysis, this trial met the requirements with a sample size ≥ 99, achieving a power of 0.987 at *n* = 240. Additionally, to evaluate the long-term reproductive outcomes of hormonal interventions, all experimental gilts were continuously tracked for subsequent mating, pregnancy, and farrowing performance.

#### Experiment on promoting estrus and ovulation in multiparous sows

This study was conducted from March to December 2024 on three large-scale conventional pig farms located in southern-central Guangdong, China. During the trial period, the average daily ambient temperature ranged from 20.0 °C to 35.0 °C, with relative humidity between 70% and 90%. The inclusion criteria were sows with similar body condition, lactation period of 24 to 26 days, and normal reproductive records; the exclusion criteria were sows suffering from reproductive system diseases, postpartum infections, obvious abnormal body condition, or eliminated due to non-experimental factors during the experiment. Based on this, 957 multiparous sows (Landrace x Large White) provided by Guangzhou Pucheng Biotechnology Co., Ltd. were randomly selected and housed in a conventional semi-intensive system. Double-blind experimental design was used during the experiment.

On the day of weaning, all sows were transferred to breeding-gestation facilities and individually housed in stalls. To closely mimic practical production conditions and minimize economic losses, only a positive control group was included in the study. The trial was implemented in the following sequential steps: (1) Optimization of spFSH dose: A total of 132 gilts were used in this part, 30 µg spFSH was finally determined to be the optimal dose (see Supplementary Material C for details) (2) Optimization of shCG dose: A total of 151 gilts were used in this part, 150 IU shCG was finally determined to be the optimal dose (see Supplementary Material D for details) (3) Comparison of Ovulation-Inducing Hormones: A total of 170 sows were allocated to receive one of the following estrus induction protocols at 24 h post-weaning: (i) the optimal spFSH + shCG combination (57 multiparous sows), (ii) PG600 [(PMSG 400 IU + hCG 200 IU)/5 ml/sow] (54 multiparous sows), or (iii) PMSG alone (1000 IU/sow) (59 multiparous sows). Sows exhibiting synchronized estrus received 200 IU shCG 72 h later to induce ovulation. FTAI was conducted with the first insemination scheduled 8 ~ 16 h after induction, followed by a second insemination 24 h later. Estrus detection was performed daily, and ovulation was monitored via ultrasonography. Pregnancy was diagnosed by ultrasound on Day 28 post-insemination, and farrowing dates and litter performance were recorded (4). Optimization of ovulation induction dose: A total of 429 sows were treated with either 1000 IU PMSG or the optimal spFSH + shCG combination at 24 h post-weaning. At 72 h post-treatment, sows were further allocated to receive one of the following ovulation inducers: hCG (500 IU/sow, Ningbo Second Hormone Factory, Zhejiang, China) (62 multiparous sows), GnRH (100 µg/sow, Ningbo Second Hormone Factory, Zhejiang, China) (62 multiparous sows), or shCG at different doses (200, 300, 400, 500, or 600 IU/sow) (Each group has 61 multiparous sows). FTAI was performed 16 h after the expected time of ovulation, followed by a second insemination 24 h later. Estrus detection, pregnancy diagnosis, and reproductive performance assessments were conducted as described above. In this trial, five sows from each treatment group (35 sows in total) in the ovulation induction experiment were selected for serial blood sampling from the anterior vena cava to monitor serum hormone levels (P_4_). Blood was collected at 13 time points: 8 h before ovulation induction (baseline) and at 24, 32, 48, 56, 72, 80, 96, 104, 120, 128, 144, and 168 h after induction (with 0 h defined as the time of hormone administration). Statistical power analysis indicated that: for the third test, a sample size ≥ 74 met the requirement, with a power of 0.983 at *n* = 170; and for the fourth test, a sample size ≥ 173 met the requirement, with a power of 0.988 at *n* = 429.

### Sow estrus detection

Starting from Day 2 after synchronized estrus induction, estrus detection was performed twice daily at the experimental pig farm, approximately 30 min after feeding. Artificial detection was carried out by one staff member spraying boar pheromone (Ningbo Second Hormone Factory, Zhejiang, China), while one to two others closely observed vulvar changes from behind the individual stalls. Sows in estrus typically exhibited signs such as decreased appetite, vulvar swelling and reddening, increased sexual receptivity, tail curling, ear erectness, a dazed expression, and the presence of viscous, stringy vulvar discharge. Upon applying back pressure, they also displayed a standing reflex accompanied by mucus discharge from the vulva [36]. Given that some gilts do not have an obvious standing reflex, the criteria for determining sow estrus are based on a comprehensive assessment: the diameter of multiple follicles must be greater than 3.5 mm and can develop to more than 4 mm [37]. At the same time, a comprehensive assessment is conducted based on a series of apparent characteristics such as obvious vulvar swelling, mucus secretion, and standing still. In contrast, estrus in multiparous sows was identified based on a positive standing reflex.

### Sow B-ultrasound test

#### Follicular monitoring and pregnancy diagnosis

Ovarian development and pregnancy were monitored by transabdominal B-mode ultrasonography (HS-1600, Honda Electronics Co., Ltd., Japan). Follicular scans were performed twice daily, 8–10 h apart, using a convex-sector probe with coupling gel applied above the penultimate teat. Full bladder visualization aided anatomical orientation, and follicle diameters were measured; follicles > 10 mm persisting ≥ 5 days were classified as cystic. Return-to-estrus was assessed 18–24 days after the final insemination using boar pheromone spray. Sows not exhibiting estrus were scanned at 28–35 days post-insemination, and the presence of round anechoic structures (embryonic vesicles) indicated pregnancy. Unclear cases were re-examined after 35 days.

#### Determination of ovulation time in weaned sows

Follicular growth was evaluated by calculating the average diameter of the two to four largest follicles on one ovary. Ovulation was considered complete when the number of large follicles (≥ 6 mm in diameter) decreased significantly or no follicles ≥ 3 mm were observed in the ultrasound field, and round anechoic structures with embryonic structures were present. Ovulation time was defined as the midpoint between the last observation and the first detection of ovulation completion, with testing performed once a day in the morning and afternoon, approximately 8–10 h apart [38].

### Artificial insemination of sows

Before insemination, the vulva and perineal area of each sow were thoroughly cleaned and disinfected. The semen used for artificial insemination was obtained from the experimental farm and carefully screened to ensure high quality, with sperm motility ≥ 80%, a total sperm count of 1.5 × 10⁹ per bag, and a volume of 60 mL. Multiparous sows were inseminated twice using the post-cervical insemination technique, which involved a flexible inner tube (15 ~ 20 cm) inserted beyond the cervical folds into the uterine body to enhance fertilization efficiency. Gilts were not inseminated during the trial and were only used for hormone treatment and estrus observation.

### Pharmacokinetic testing

To compare the pharmacokinetic profiles of shCG and uhCG in sows, a randomized, single-dose, open-label, two-period, two-treatment crossover design with a 21-day washout period was employed. The study was conducted by Beijing VJTBio Co., LTD, which also provided the analytical data. A total of 26 healthy adult sows were enrolled and randomly assigned to two groups (*n* = 13). The test group received 1000 IU shCG via intramuscular injection in the neck, while the reference group received 1000 IU uhCG. Blood samples were collected at designated time points, and serum drug concentrations were determined (1 h、4 h、8 h、12 h、24 h、48 h、72 h、96 h、120 h、144 h、168 h).

For spFSH, a randomized, single-dose, open-label design was used. This study was also performed by Beijing VJTBio Co., LTD, which provided the corresponding data. Adult sows received either intramuscular injections of spFSH at doses of 0.25 µg/kg (IM-L), 0.5 µg/kg (IM-M), or 1.0 µg/kg (IM-H), or a single intravenous injection of 0.5 µg/kg (IV). Blood samples were collected for pharmacokinetic analysis of serum drug concentrations (0, 0.5, 1, 2, 4, 8, 12, 24, 36, 48, 72, 96, 120, 144, 168 and 216 h).

### Collection and determination of serum hormones

Blood samples were collected from the anterior vena cava, centrifuged at 4000 r/min for 10 min to obtain serum, which was aliquoted, stored at − 20 °C, and transported on dry ice to Beijing North Biotechnology Institute Co., Ltd. (Beijing, China). Serum E2, FSH, and LH concentrations were determined by radioimmunoassay, and P4 was measured by ELISA at 450 nm.

### Sow breeding data collection and calculation method

The data collected following the puberty induction study focused on the first insemination performed after the experiment ended, when the gilts reached a certain age and joined the herd. Comprehensive production data were systematically collected from insemination to farrowing, including age at herd entry, insemination dates, pregnancy diagnosis results, farrowing dates, total litter size, number of viable piglets, and culling dates prior to farrowing.

For the trial on estrus promotion and ovulation induction in multiparous sows, data collected included the previous weaning date, farrowing date, culling date due to breeding failure, as well as relevant farrowing parameters such as total litter size and number of viable piglets. All data were manually recorded onsite to ensure authenticity and completeness of critical timepoints. The calculation method of important indicators is the same as [39].

### Statistical analysis of data

All data were initially organized and preprocessed in Excel 2021 (Microsoft, Redmond, WA, USA). Subsequently, statistical significance was analyzed using generalized linear mixed models (GLMM) implemented in IBM SPSS^®^ Statistics 26 (IBM, Armonk, NY, USA). Graphs were generated using GraphPad Prism 9.5.0 (GraphPad Software, San Diego, CA, USA). Results are presented as mean ± standard error (Mean ± SE) or percentages (%). Statistical significance was defined at *P* < 0.05.

For the puberty induction trial in gilts, the GLMM included treatment as a fixed effect and trial batch as a random effect. Response variables comprised estrus rate within 7 days, incidence of follicular cysts, total litter size per litter, and number of viable piglets per litter. In the estrus promotion and ovulation induction trials for multiparous sows, treatment was included as a fixed effect, while trial batch and parity were modeled as random effects. Response variables included ovulation timing, estrus rate, follicular development, follicular cyst incidence, conception rate, farrowing rate, and litter performance parameters such as total litter size. The significance analysis of serum test results showed that E_2_, FSH, and LH levels followed normal distribution after normality test, so two-way analysis of variance was used; however, P_4_ level did not follow normal distribution, so non-parametric test was used for analysis. Specifically, comparisons among groups were performed using the Kruskal–Wallis test, post hoc comparisons were conducted with Dunn’s test followed by Bonferroni correction, and time effects were analyzed using the Friedman test.

## Results

### Pharmacokinetic effects of spFSH and shCG

The shCG exhibited favorable pharmacodynamic persistence and systemic exposure in sows. While its mean half-life was comparable to that of urinary-derived hCG (uhCG), shCG demonstrated a higher peak concentration and shorter time to peak, suggesting advantages in onset and potency (Supplementary Material E).

The spFSH showed slow absorption, prolonged duration, and robust systemic exposure following intramuscular injection. Compared to natural FSH preparations (half-life: 2 ~ 4 h), spFSH had extended half-lives and mean residence times exceeding 66 h across all intramuscular doses, indicating sustained activity during key pre-ovulatory stages and supporting synchronized follicular development (Supplementary Material F).

### Screening for the optimal combination of spFSH and shCG to induce puberty in gilts

#### Synergistic effect of spFSH + shCG on puberty induction and ovarian cyst

To evaluate the differences in the efficacy of different hormone products in inducing sexual maturation, a comparative trial was conducted using different treatment regimens after the optimal spFSH & shCG combination was successfully selected (see Supplementary Materials A and B). As shown in Table 1, compared with the Control group, the PMSG group, 30 µg spFSH & 150 IU shCG group, and PG600 group exhibited significantly higher estrus rates within 7 days post-treatment (*P* < 0.05). However, the incidence of ovarian cysts was significantly higher in the PMSG and PG600 groups than in the 30 µg spFSH & 150 IU shCG and Control groups (*P* < 0.05). These results indicate that the combination of 30 µg spFSH and 150 IU shCG effectively promotes puberty onset in gilts while significantly reducing the risk of ovarian cyst formation, demonstrating application potential.

**Table 1:** Comparative test of products for inducing first estrus in gilts

Items	Groups				*P*-Value
	Control	PMSG	30 µg spFSH & 150 IU shCG	PG600	
Dosage	Saline	800 IU PMSG	30 µg spFSH + 150 IU shCG	5 ml PG600^1^	/
Number of gilts	77	62	77	77	/
Number of gilts in estrus	24	51	59	61	/
Estrus rate (%)	31.17%^a^	82.26%^b^	76.62%^b^	79.22%^b^	< 0.001
Number of gilts with follicular cysts	1	9	5	19	/
Follicular cyst rate (%)	1.30%^a^	14.52%^b^	6.49%^a^	24.68%^b^	< 0.001

Note: Except for the hormone treatments during the experimental phase, all subsequent estrus, ovulation, and insemination procedures followed standard farm protocols. ^1^5ml PG600 includes 400 IU PMSG and 200 IU hCG. ^a, b^Different superscripts within rows differ significantly (*P* < 0.05)

#### Application of SpFSH combined with ShCG on reproductive efficiency

In this study, farrowing data were collected from 175 of 293 enrolled gilts due to ear tag loss and other factors (Table 2). No significant differences were observed among treatment groups in total or qualified piglets per litter. The 30 µg spFSH & 150 IU shCG group exhibited the higher efficiency, reducing “Breeding female inventory days per piglet born” by 4.38 days and “Breeding female inventory days per qualified piglet born” by 2.05 days compared to the control. The PMSG group saved 1.07 and 0.27 days, respectively, while PG600 saved 1.62 days for piglets born but increased qualified piglet days by 0.5.

**Table 2:** Effects of different hormonal treatments on the utilization efficiency of gilts

Items	Groups				*P*-Value
	Control	PMSG	30 µg spFSH & 150 IU shCG	PG600	
Dosage	Saline	800 IU PMSG	30 µg rpFSH + 150 IU rhCG	5 ml PG600^1^	/
Number of gilts	49	41	45	40	/
Total number of farrowing gilts	46	38	45	37	/
Number of gilts leaving the farm	3	3	0	3	/
Total number of piglets per litter	11.04 ± 3.14	11.56 ± 2.87	12.80 ± 4.15	11.48 ± 3.79	0.723
Average number of qualified piglets per litter	10.06 ± 2.82	10.27 ± 2.33	10.71 ± 3.16	9.80 ± 2.82	0.459
Breeding female inventory days per piglet born (d/head)	32.64	31.57	28.26	31.02	/
Breeding female inventory days per qualified piglet born (d/head)	35.82	35.55	33.77	36.32	/

Note: Total number of piglets per litter = the total number of piglets born by sows in one litter / the number of sows entering the group

Average number of qualified piglets per litter = the number of qualified piglets born by sows in one litter / the number of sows entering the group

Breeding female inventory days per piglet born = (the age of sows born in one litter + the age of sows leaving the breeding farm without giving birth) / the total number of piglets born by sows in one litter

Breeding female inventory days per qualified piglet born = (the age of sows born in one litter + the age of sows leaving the breeding farm without giving birth) / the number of qualified piglets born by sows in one litter

^1^5ml PG600 includes 400 IU PMSG and 200 IU hCG

#### Effects of different hormones on sex hormones in gilts during puberty induction

**Fig. 1:**
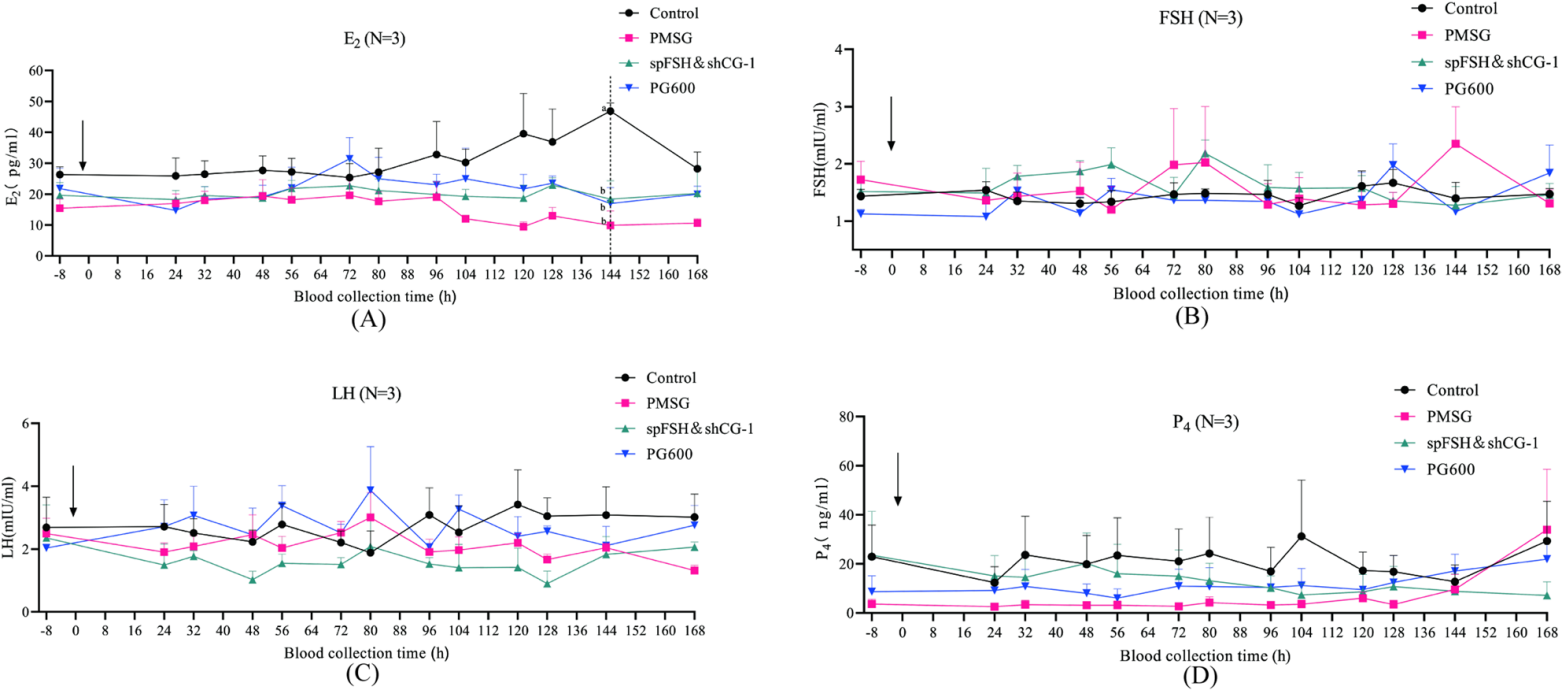
Hormonal responses to various puberty induction regimens in gilts (*n* = 3 per group). (**A**) Temporal changes in E₂ levels across groups at 13 sampling points. (**B**) Temporal changes in FSH levels across groups at 13 sampling points. (**C**) Temporal changes in LH levels across groups at 13 sampling points. (**D**) Temporal changes in P₄ levels across groups at 13 sampling points. All data are expressed as mean ± SEM

Given that the onset of puberty is closely associated with fluctuations in hormone levels, serum samples were collected from gilts at 13 time points (8 h before treatment and 24, 32, 48, 56, 72, 80, 96, 104, 120, 128, 144, and 168 h after treatment) to monitor the dynamic changes in E_2_, FSH, LH, and P_4_ levels. The results showed no significant differences in FSH, LH, or P_4_ levels among the treatment groups, and this pattern remained consistent throughout the experimental period. However, at 144 h, a significant difference was observed in E_2_ levels, with the CON group exhibiting a significantly higher E_2_ concentration than the other three groups (*P* < 0.05) (Fig. 1). These findings indicate that hormone treatment allows gilts to reach hormone levels comparable to those of naturally cycling animals at estrus.

### Screening of optimal spFSH + shCG combination for inducing estrus and ovulation in multiparous sows

#### Application of spFSH combined with shCG to optimize estrus synchronization and reproductive efficiency in sows

##### spFSH + shCG regulate follicular development and cyst formation

To evaluate the effects of different hormonal products on estrus promotion in multiparous sows, a comparative trial was conducted using various treatment protocols (after determining the optimal combination of spFSH and shCG; details are provided in Supplementary Materials C and D). In this trial, estrus synchronization was performed on 170 sows at 24 h post-weaning using several hormonal regimens. The results showed no significant differences in estrus and conception rates among groups (*P* > 0.05; Table 3). However, the incidence of follicular cysts in the PG600 group was significantly higher than that in the 30 µg spFSH & 150 IU shCG group and the Control group (*P* < 0.05).

**Table 3:** Comparison of estrus and fertility responses to various promotion protocols in multiparous sows

Items	Groups			*P*-Value
	Control	30 µg spFSH & 150 IU shCG	PG600	
Promote estrus	1000 IU PMSG	30 µg spFSH + 150 IU shCG	5 ml PG600^1^	/
Number of sows	59	57	54	/
Number of sows in estrus	59	57	54	/
Estrus rate (%)	100.00%	100.00%	100.00%	/
Number of pregnant sows	48	48	41	/
Conception rate (%)	81.36%	84.21%	75.93%	0.541
Number of sows	20	20	22	/
Sows with follicular cysts	2	0	5	/
Follicular cyst rate (%)	10.00%^a^	0.00%^a^	22.73%^b^	0.045

Note: In this experiment, shCG was used for ovulation induction at a dose of 200 IU. ^1^5ml PG600 includes 400 IU PMSG and 200 IU hCG. ^a, b^Different superscripts within rows differ significantly (*P* < 0.05)

##### Impact of spFSH + shCG on reproductive efficiency

As shown in Table 4, the 30 µg spFSH & 150 IU shCG group significantly increased the total number of piglets born and the number of qualified piglets compared to the PG600 group (*P* < 0.05), with values also higher than those of the Control group (*P* > 0.05). For the number of days each piglet requires to be raised by a sow, the 30 µg spFSH & 150 IU shCG group outperformed the others, followed by the Control and PG600 groups, with the Control and 30 µg spFSH & 150 IU shCG groups saving 1.08 and 1.31 days, respectively, per additional piglet compared to the PG600 group. For the number of days each qualified piglet requires to be raised by a sow, the Control group was optimal, followed by 30 µg spFSH & 150 IU shCG and PG600, saving 1.36 and 1.31 days, respectively, compared to PG600.

**Table 4:** Effects of estrus-inducing products on farrowing and sow utilization

Items	Groups			*P*-Value
	Control	30 µg spFSH & 150 IU shCG	PG600^1^	
*n*	59	57	54	/
Farrowing sows	48	45	40	/
Sows leaving the farm	11	12	14	/
The number of days each piglet requires to be raised by a sow (d/head)	10.5	10.27	11.58	/
The number of days each qualified piglet requires to be raised by a sow (d/head)	11.13	11.18	12.49	/
Total litter size	12.27 ± 1.97^ab^	12.87 ± 2.54^a^	11.65 ± 2.24^b^	0.027
Number of live pigs	11.92 ± 2.27	12.27 ± 2.57	11.23 ± 2.92	0.159
Number of qualified piglets	11.58 ± 1.90^ab^	11.84 ± 2.60^a^	10.80 ± 2.52^b^	0.034
Number of weak piglets	0.31 ± 0.82	0.40 ± 0.95	0.43 ± 0.95	0.819
Number of mummies	0.02 ± 0.14	0.04 ± 0.21	0.08 ± 0.35	1.000
Number of stillbirths	0.33 ± 0.59	0.56 ± 1.31	0.35 ± 1.30	1.000

Note: The number of days each piglet requires to be raised by a sow = (Days from last weaning to next farrowing + Days from last weaning to culling without farrowing)/Total number of piglets born; The number of days each qualified piglet requires to be raised by a sow = (Days from the previous weaning to the next farrowing + Days from the previous weaning to culling without farrowing)/Number of qualified piglets born. ^a, b^Different superscripts within rows differ significantly (*P* < 0.05).^1^5ml PG600 includes 400 IU PMSG and 200 IU hCG

#### Optimal ShCG dose for inducing synchronous ovulation and improving reproductive efficiency in multiparous sows

##### Comparison of different hormone regimens for ovulation induction

Based on the optimal estrus-promoting combination (30 µg spFSH + 150 IU shCG), this study further evaluated the optimal shCG dose (200, 300, 400, 500, 600 IU) for inducing synchronized ovulation in multiparous sows, in comparison with commonly used hormonal protocols. Estrus synchronization was initiated 24 h after weaning. As shown in Table 5, estrus rates did not differ significantly among groups (*P* > 0.05), but the conception rate in the F2 group (300 IU shCG) was significantly different from that of the 200 IU, 400 IU, and 600 IU groups (*P* < 0.05), indicating that 300 IU is the optimal dose for inducing ovulation in multiparous sows.

**Table 5:** Optimal ShCG ramp test for inducing synchronized ovulation in multiparous sows: estrus and conception

Items		Groups							*P*-Value
		PMSG + hCG	PMSG + GnRH	F1	F2	F3	F4	F5	
Hormone dosage	Promote estrus	1000 IU PMSG		30 µg spFSH + 150 IU shCG					/
	Inducing ovulation	500 IU hCG	100 µg GnRH	200IU shCG	300IU shCG	400IU shCG	500IU shCG	600IU shCG	/
Number of sows		62	62	61	61	61	61	61	/
Number of estrus before ovulation induction		57	57	57	55	57	59	56	/
Post-weaning estrus rate (%)		91.94%	91.94%	93.44%	90.16%	93.44%	96.72%	91.80%	0.616
Number of pregnant sows		55	54	53	57	52	54	52	/
Conception rate (%)		88.71%^ab^	87.10%^ab^	86.89%^a^	93.44%^b^	85.25%^a^	88.52%^ab^	85.25%^a^	0.031
Ovulation within 16 h (%)		23.08%	38.46%	46.15%	15.39%	38.46%	38.46%	38.46%	/
Ovulation occurs within 16–40 h (%)		69.23%	61.54%	38.46%	69.23%	46.15%	61.54%	53.85%	/
Ovulation occurs 40 h later (%)		7.69%	0	15.39%	15.39%	15.39%	0	0	/

Note: ^a, b^Different superscripts within rows differ significantly (*P* < 0.05)

##### Comparative analysis of hormonal regimens on reproductive performance in multiparous sows

A total of 429 multiparous sows were enrolled in the trial, with 71 sows excluded due to breeding failure or removal. As shown in Table 6, no significant differences were observed among treatment groups in farrowing performance (*P* > 0.05). To further assess economic efficiency, two indicators were analyzed: “the number of days each piglet requires to be raised by a sow” and “the number of days each qualified piglet requires to be raised by a sow.” The F2 group achieved the best performance in the former (7.84 days per piglet), while the F1 group showed the greatest efficiency in the latter (9.23 days per piglet). Overall, all treatment groups exhibited improved sow utilization compared to the PMSG + hCG group.

**Table 6:** Optimal shCG ramp test results for inducing synchronous ovulation in multiparous sows

Items	Groups							*P*-Value
	PMSG + hCG	PMSG + GnRH	F1	F2	F3	F4	F5	
*n*	62	62	61	61	61	61	61	/
Farrowing sows	48	52	54	55	49	49	51	/
The number of days each piglet requires to be raised by a sow (d/head)	8.89	8.19	7.88	7.84	8.58	8.61	8.22	/
The number of days each qualified piglet requires to be raised by a sow (d/head)	10.42	9.69	9.23	9.47	10.10	10.17	9.58	/
Total litter size	14.41 ± 2.97	14.96 ± 3.38	15.19 ± 2.71	15.35 ± 3.85	14.54 ± 3.20	14.60 ± 3.24	14.88 ± 3.08	0.764
Number of live pigs	13.22 ± 3.03	13.52 ± 3.21	13.96 ± 2.54	13.83 ± 3.39	13.21 ± 2.83	13.08 ± 3.35	13.66 ± 2.82	0.715
Number of qualified piglets	12.33 ± 2.62	12.66 ± 2.89	12.96 ± 2.03	12.7 ± 3.02	12.35 ± 2.46	12.35 ± 3.07	12.76 ± 2.39	0.861
Number of weak piglets	1.2 ± 1.68	1.44 ± 1.78	1.23 ± 1.63	1.55 ± 1.73	1.33 ± 1.67	1.52 ± 3.3	1.22 ± 1.69	0.576
Number of mummies	0.28 ± 0.67	0.22 ± 0.72	0.21 ± 0.56	0.24 ± 0.6	0.21 ± 0.61	0.15 ± 0.46	0.08 ± 0.27	0.720
Number of stillbirths	0.72 ± 1.03	0.76 ± 1.12	0.83 ± 1.04	1.02 ± 1.08	0.65 ± 1.09	0.63 ± 0.9	0.82 ± 1.14	0.962

#### Effects of different hormone regimens on the dynamic changes of P_4_ levels in sows

**Fig. 2:**
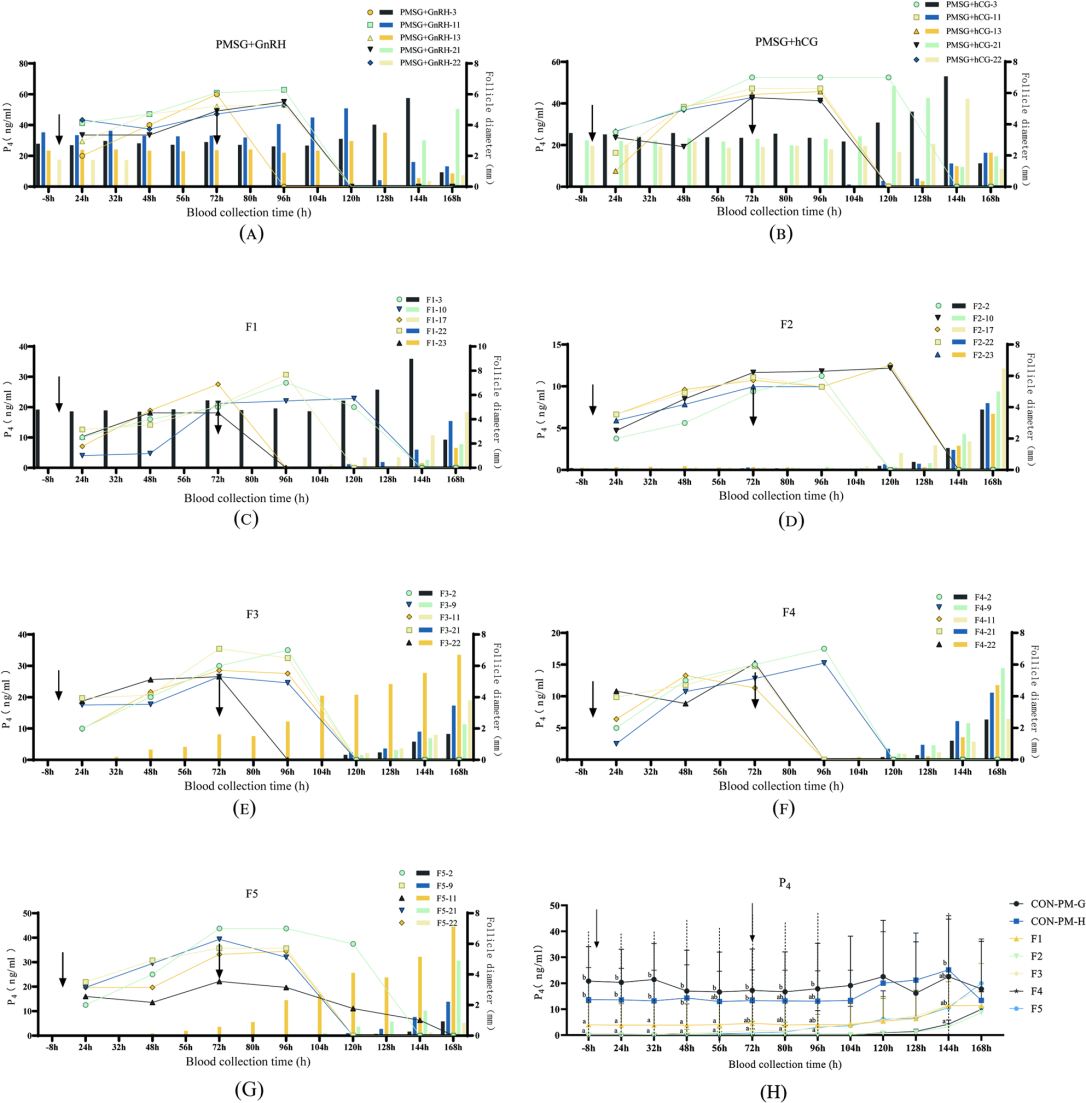
Effects of each group in the experiment of inducing synchronous ovulation in multiparous sows on the serum P_4_ level of sows (*n* = 5 per group). Note: Figures (**A** ~ **G**) illustrate the changes in P_4_ levels at 13 time points: 8 h before treatment and 24, 32, 48, 56, 72, 80, 96, 104, 120, 128, 144, and 168 h after treatment. Figures H refer to the trends of P_4_ levels in each group. The bar graphs represent serum P_4_ concentrations (left Y-axis), while the line graphs depict the average follicle diameter of sows on the corresponding blood collection days (right Y-axis). The downward arrows (↓) indicate the time of estrus induction or ovulation induction treatment. In the figure, F3-22, F4-22, and F5-11 are non-pregnant sows, and the rest are successfully pregnant; F2-2, F2-23, F3-22, and F5-11 were not in estrus when ovulation was induced (72 h), and the rest were in estrus

To investigate the effects of different hormonal treatments on endogenous steroid hormone levels, this study systematically monitored serum P_4_ dynamics in sows across treatment groups. Results revealed a clear trend of increased P_4_ levels following synchronized ovulation across all groups. However, the PMSG + GnRH group (Fig. 2A) showed aberrantly high P_4_ levels during the follicular phase in most sows (except PMSG + GnRH-21 and − 22), with a subsequent decline after ovulation in those with elevated pre-ovulatory P_4_. In the PMSG + hCG group (Fig. 2B), a similar pattern was noted: except for PMSG + hCG-11 and − 13, most sows had high P_4_ during the follicular phase, with a subsequent decline after ovulation. In contrast, sows in the F1 ~ F5 groups (Fig. 2C ~ G) maintained low P_4_ levels during the follicular phase, followed by a marked and sustained rise within 48 h after synchronized ovulation. Notably, individuals such as F1-3, F3-22, and F5-11 exhibited an earlier onset of P_4_ elevation, and F1-3 showed a distinct post-ovulatory drop thereafter. In general, the levels in CON-PM-G and H were higher than those in the other five groups at and before 144 h; however, both groups exhibited a decreasing trend at 168 h (Fig. 2H).

## Discussion

Currently, hormonal products widely used in sow reproduction, such as FSH, PMSG, PG600, and GnRH, exhibit limitations including short half-life [40], unstable sources [41], high ovarian cyst incidence [42–44], and low reliability [7, 45, 46]. In contrast, spFSH and shCG, produced via CHO cell expression, provide longer half-life and stable sources, indicating strong application potential [17, 47]. However, systematic research on their combined efficacy is limited. This study evaluated the effects of spFSH combined with shCG on inducing puberty in gilts and promoting estrus and ovulation in multiparous sows, compared with traditional hormonal protocols, to better understand its efficacy in improving reproductive performance.

### Different hormone regimens to induce puberty in gilts

The onset of puberty plays a pivotal role in determining the subsequent reproductive performance of replacement gilts. Our results indicate that PMSG, PG600, and the combination of 30 µg spFSH + 150 IU shCG can effectively promote the onset of puberty in gilts. Notably, compared with PMSG and PG600, the 30 µg spFSH + 150 IU shCG combination more effectively controls the risk of ovarian cyst formation.

Our findings are generally consistent with previous reports on the use of PMSG and PG600 to induce puberty in gilts [17, 48–50]. Subsequent studies further demonstrated that the combined administration of rFSH and hCG significantly increased the 7-day estrus rate in gilts [51–53]. Similarly, two independent studies found that 150-day-old gilts treated with extracted FSH and hCG showed an estrus rate of 64%, which rose to 85% when rFSH and hCG were used in combination [17, 54]. These differences may be due to differences in sow age and sexual maturity, as well as the pharmacokinetic properties of rFSH, such as its prolonged half-life and higher bioactivity [43, 55].

Ovarian cysts are a common constraint on sow reproductive performance, often resulting from hypothalamic–pituitary–ovarian (HPO) axis dysfunction or insufficient LH secretion, leading to impaired ovulation, reduced litter sizes, and compromised fertility [56, 57]. Traditional hormonal agents, such as PMSG and PG600, have been reported to increase follicular cyst incidence [9, 42–45, 52, 58], consistent with our findings that the PG600 and PMSG groups exhibited higher cyst rates than the control or 30 µg spFSH + 150 IU shCG groups. This effect may stem from low endogenous hormone levels and an immature HPO axis prior to puberty, where excessive FSH/LH-like activity could disrupt endocrine balance, triggering abnormal follicular growth or atresia and increasing cyst formation risk [59]. In contrast, the combination of 30 µg spFSH and 150 IU shCG not only increased the estrus rate but also effectively reduced the incidence of cysts, demonstrating better ovarian regulation function. While the precise mechanisms remain elusive, they may involve receptor-binding specificity and differential modulation of the endocrine axis, meriting further exploration.

### Effects of different hormone regimens on reproductive performance of multiparous sows

Gonadotropins have been used in weaned sows to effectively stimulate follicular development and promote estrus synchronization [1, 35, 60]. However, to our knowledge, research on the combined application of spFSH and shCG in porcine reproductive production is still very limited. In this study, we evaluated the efficacy of PMSG, PG600, and a combination of 30 µg spFSH + 150 IU shCG on estrus induction in multiparous sows and further compared the role of conventional ovulation induction protocols with shCG in ovulation regulation. Our results demonstrate that PMSG, PG600, and a combination of 30 µg spFSH and 150 IU shCG are effective in inducing estrus in multiparous sows. It is noteworthy that compared with PMSG or PG600, combined treatment with spFSH + shCG better controlled cystogenesis, indicating that it may have a better effect in maintaining ovarian function regulation. Furthermore, after estrus stimulation with this combination, administration of 300 IU shCG for ovulation induction further improved conception rates.

Our results are consistent with previous findings showing that hormonal treatments effectively promote estrus in multiparous sows [17, 61, 62]. Similarly, in agreement with earlier studies [17], no significant differences in estrus and conception rates were observed between treatments using synthetic hormones and those using PMSG. Notably, comparative studies between synthetic gonadotropins and PG600 remain limited, and further research is needed to clarify their relative efficacy and reproductive outcomes in sows. The incidence of ovarian cysts was significantly higher in the PG600 group, aligning with previous results [63, 64]. Ovarian cysts can impair reproductive performance by disrupting the intra-follicular microenvironment, which inhibits follicular development, accelerates atresia, and reduces ovulation rates, thereby decreasing the number of viable follicles and compromising both ovulation efficiency and embryonic developmental potential [57, 65, 66]. In this study, the PG600 group also exhibited significantly lower total piglets born and qualified piglets compared to the 30 µg spFSH & 150 IU shCG group, further confirming the negative impact of ovarian cysts on sow fertility. These results suggest that, compared with traditional hormone preparations, 30 µg spFSH & 150 IU shCG offers comparable performance in inducing estrus in multiparous sows, but with better control of cyst incidence, making it more suitable for intensive pig production systems.

We further compared shCG with traditional hormonal regimens for ovulation induction. Treatment with 30 µg spFSH & 150 IU shCG to induce estrus, followed by 300 IU shCG at 72 h post-synchronization and insemination at 16 h and 40 h, achieved a conception rate of 93.44%, which are slightly higher than those reported in previous studies [17]. In contrast, using the same insemination protocol with estrus induction by PMSG followed by ovulation induction with GnRH or hCG resulted in lower conception rates of 87.10% and 88.71%, respectively, which are still slightly higher than previously reported values [23, 67]. The slightly higher values compared with previous studies may be attributed to differences in the insemination protocols, while the observed variations in this study could be explained by differences in the mechanisms of hormone action. Specifically, shCG directly binds to LHCGR on granulosa and luteal cells, mimicking a sustained LH-like surge that promotes follicular maturation, oocyte meiotic resumption, and corpus luteum formation [32–34]. This direct action provides more consistent ovulatory responses than GnRH, which depends on endogenous LH release and can be influenced by factors such as sow parity, seasonal anestrus, or pituitary LH reserves [23, 24]. Additionally, compared with uhCG, shCG offers superior purity, molecular stability, and batch-to-batch consistency, reducing degradation and variability that may compromise oocyte quality and bioactivity [3, 31]. Serum analysis showed that sows in the spFSH + shCG group maintained a physiological P_4_ pattern, with low levels during the follicular phase and steadily increasing levels after ovulation, reflecting the coordination of ovarian and uterine function. These results suggest that the combination of 30 µg spFSH & 150 IU shCG likely provides stable follicle-stimulating signals in the early follicular phase, followed by accurately timed ovulation induction via shCG, enabling tight ovulation synchrony and optimal utilization of the fertilization window [14, 18, 68].

### Limitations of this study

Although these findings are promising, several considerations should be noted when interpreting these results.

Although we performed sample size calculations and power analyses to ensure statistical reliability, funding limitations limited us to a minimum sample size, and the number of animals in some treatment groups was limited. In addition, this study was not validated in individuals with different genetic backgrounds, which may affect the generalizability of the results. Second, although differences in estrous response and cyst incidence were detected among different hormone treatments, the underlying physiological and molecular mechanisms remain unclear and require further elucidation. In addition, due to funding limitations, this study focused only on the reproductive outcomes of the first parity after hormone treatment and did not assess subsequent reproductive cycles or lifetime productivity. On the other hand, the potential immunogenicity of spFSH and shCG should also be explored. Although spFSH, composed of porcine FSH and Fc, may be less immunogenic than PMSG, both it and hCG, as a foreign protein in sows, have the potential to elicit neutralizing antibody responses [69].

Therefore, future studies should include larger sample sizes and multiple genotypes, investigate the dynamics of follicular reserve and overall reproductive lifespan in treated sows, and comprehensively evaluate the immunogenic response to recombinant hormone administration.

## Conclusion

This study systematically evaluated the value of combining spFSH with shCG in porcine reproductive management and provided a novel FTAI protocol. Our results showed that 30 µg spFSH combined with 150 IU shCG could advance the onset of puberty in gilts and promote post-weaning estrus in multiparous sows, while controlling the incidence of follicular cysts and improving the uniformity of follicular development. Furthermore, administering 300 IU of shCG 72 h after estrus synchronization to promote ovulation in multiparous sows, followed by breeding 16 and 40 h later, improved conception rates and overall reproductive efficiency. Future studies are necessary to further elucidate the potential molecular regulatory mechanisms of different hormone actions and optimize hormone intervention strategies.

## Supplementary Information

Below is the link to the electronic supplementary material.

Supplementary Material 1

## Data Availability

The original contributions presented in the study are included in the article/supplementary material, further inquiries can be directed to the corresponding authors.
