# Supplementary material for: Effects of synthetized porcine follicle-stimulating hormone and synthetized human chorionic gonadotropin on reproductive efficiency in pigs
Source: Porcine Health Manag. 2025 Dec 29;11:65. doi: 10.1186/s40813-025-00476-z (PMC12751420; doi:10.1186/s40813-025-00476-z)
Supplement: Supplementary file 1 — Supplementary Material 1 [file 40813_2025_476_MOESM1_ESM.docx]

Appendix A spFSH dosage screening for gilts’ first estrus induction

At 24 hours after pen transfer, 108 prepubertal gilts that had not exhibited estrus were intramuscularly injected with different doses of spFSH (20, 30, or 40 μg; Beijing VJTBio Co., LTD, Beijing, China) combined with a fixed dose of shCG (200 IU; Beijing VJTBio Co., LTD, Beijing, China). The control group (36 prepubertal gilts) received 5ml physiological saline (Shaanxi Shengao Animal Pharmaceutical Co., Ltd., Shaanxi, China). Estrus detection and ultrasound examination were performed daily after treatment to determine the optimal dose of spFSH for inducing puberty onset in gilts. According to statistical power analysis, this trial met the requirements with a sample size ≥99, reaching a power of 0.912 at n = 144.

| Items | Groups | | | | *P*-Value |
| --- | --- | --- | --- | --- | --- |
|  | Control | A1 | A2 | A3 |  |
| Hormone Dosage | Saline  + Saline | 20 μg spFSH  +200 IU shCG | 30 μg spFSH  +200 IU shCG | 40 μg spFSH  +200 IU shCG | / |
| Number of gilts | 36 | 36 | 36 | 36 | / |
| Number of gilts in estrus | 5 | 18 | 29 | 25 | / |
| Estrus rate (%) | 13.89%^a^ | 50.00%^b^ | 80.56%^c^ | 69.44%^c^ | <0.001 |

Note: ^a,b,c^Different superscripts within rows differ significantly (*P* < 0.05).

Appendix B. shCG dosage screening for gilts’ first estrus induction

Based on the optimal spFSH (30 μg) dose determined in Appendix C, a second trial was conducted in 175 prepubertal gilts. At 24 hours post-transfer, gilts were intramuscularly administered the optimal spFSH dose in combination with varying doses of shCG (100, 150, 200, 250, or 300 IU), while the control group (35 prepubertal gilts) again received 5ml physiological saline. Estrus detection and ultrasound examination were performed daily after treatment to determine the optimal dose of spFSH for inducing puberty onset in gilts. According to statistical power analysis, this trial met the requirements with a sample size ≥148, with a power of 0.905 at n = 210.

| Items | Groups | | | | | | *P*-Value |
| --- | --- | --- | --- | --- | --- | --- | --- |
|  | Control | B1 | B2 | B3 | B4 | B5 |  |
| Hormone Dosage | Saline  + Saline | 30 μg spFSH  +100 IU shCG | 30 μg spFSH  +150 IU shCG | 30 μg spFSH  +200 IU shCG | 30 μg spFSH  +250 IU shCG | 30 μg spFSH  +300 IU shCG | / |
| Number of gilts | 35 | 35 | 35 | 35 | 35 | 35 | / |
| Number of gilts in estrus | 7 | 11 | 27 | 27 | 23 | 28 | / |
| Estrus rate（%） | 20.00%^a^ | 31.43%^a^ | 77.14%^b^ | 77.14%^b^ | 65.71%^b^ | 80.00%^b^ | <0.001 |

Note: ^a,b^Different superscripts within rows differ significantly (*P* < 0.05).

Appendix C. Optimal spFSH ramping test to promote estrus in multiparous sows

A total of 99 multiparous sows were intramuscularly injected with different doses (20, 30, or 40 μg) of spFSH plus 200 IU shCG at 24 h post-weaning. The control group (33 multiparous sows) received 1000 IU of PMSG. All sows that exhibited standing estrus were administered an additional 200 IU shCG intramuscularly to induce ovulation. A TAI protocol was followed: the first insemination occurred 24 h after the onset of standing estrus, followed by a second insemination 16~24 h later. Estrus detection was performed daily, and ovulation was monitored via ultrasonography. Pregnancy was diagnosed by ultrasound on Day 28 post-insemination, and farrowing dates and litter performance were recorded. Statistical power analysis indicated that: for this test, a sample size ≥99 met the requirement, with a power of 0.890 at n = 132.

| Items | Groups | | | | *P*-Value |
| --- | --- | --- | --- | --- | --- |
|  | Control | D1 | D2 | D3 |  |
| Promote estrus | 1000 IU PMSG | 20 μg spFSH  +200 IU shCG | 30 μg spFSH  +200 IU shCG | 40 μg spFSH  +200 IU shCG | / |
| Number of sows | 33 | 33 | 33 | 33 | / |
| Number of sows in estrus | 33 | 33 | 33 | 33 | / |
| Estrus rate (%) | 100.00% | 100.00% | 100.00% | 100.00% | / |
| Number of pregnant sows | 31 | 24 | 27 | 24 | / |
| Conception rate (%) | 93.94%^a^ | 72.73%^b^ | 81.82%^ab^ | 72.73%^b^ | 0.021 |
| Interval from estrus treatment to standing still (h) | 71.04±17.37 | 70.56±13.29 | 75.60±15.33 | 70.32±11.50 | 0.762 |
| Diameter of standing follicle (mm) | 6.38±4.94 | 5.24±0.57 | 5.32±0.98 | 5.12±0.88 | 0.550 |
| Interval from synchronized ovulation treatment to the end of ovulation (h) | 46.8±26.16 | 40.08±20.07 | 34.8±17.64 | 43.92±23.34 | 0.583 |

Note: In this experiment, shCG was used for ovulation induction at a dose of 200 IU. ^a,b,c^Different superscripts within rows differ significantly (*P* < 0.05).

Appendix D. Optimal shCG ramping test for promoting estrus in multiparous sows

Optimization of shCG dose: A total of 121 multiparous sows received the optimal spFSH dose determined from step (1), combined with varying doses of shCG (50, 100, 150, or 200 IU) at 24 h post-weaning. The control group (30 multiparous sows) received 1000 IU PMSG. As in step (1), all standing sows were administered 200 IU shCG to induce ovulation. TAI was conducted with the first insemination scheduled 8~16 h after the onset of standing estrus and the second 24 h later. Estrus detection was performed daily, and ovulation was monitored via ultrasonography. Pregnancy was diagnosed by ultrasound on Day 28 post-insemination, and farrowing dates and litter performance were recorded. Statistical power analysis indicated that: for this test, a sample size ≥124 met the requirement, with a power of 0.865 at n = 151;

| Items | Groups | | | | | *P*-Value |
| --- | --- | --- | --- | --- | --- | --- |
|  | Control | E1 | E2 | E3 | E4 |  |
| Promote estrus | 1000 IU PMSG | 30 μg spFSH  +50 IU shCG | 30 μg spFSH  +100 IU shCG | 30 μg spFSH  +150 IU shCG | 30 μg spFSH  +200 IU shCG | / |
| Number of sows | 30 | 30 | 30 | 31 | 30 | / |
| Number of sows in estrus | 30 | 30 | 30 | 31 | 30 | / |
| Estrus rate (%) | 100.00% | 100.00% | 100.00% | 100.00% | 100.00% | / |
| Number of pregnant sows | 24 | 20 | 22 | 27 | 24 | / |
| Conception rate (%) | 80.00%^ab^ | 66.67%^a^ | 73.33%^ab^ | 87.10%^b^ | 80.00%^ab^ | 0.038 |
| Interval from estrus treatment  to standing still (h) | 76.00±17.94 | 77.53±17.20 | 72.97±16.15 | 73.23±21.80 | 73.07±16.21 | 0.815 |
| Diameter of standing follicle (mm) | 5.37±0.66 | 4.72±0.81 | 4.98±0.96 | 4.65±0.90 | 4.72±0.44 | 0.706 |
| Interval from synchronized ovulation treatment to the end of ovulation (h) | 26.47±18.03^a^ | 42.62±14.71^ab^ | 48.71±25.42^b^ | 43.23±16.33^ab^ | 32.44±16.87^ab^ | 0.178 |

Note: In this experiment, shCG was used for ovulation induction at a dose of 200 IU. ^a,b^Different superscripts within rows differ significantly (*P* < 0.05).

Appendix E: Pharmacokinetic data for shCG

A randomized single-dose, open, double-period, double-treatment crossover design was used, with a washout period of 21 days between two doses. 26 adult sows (100~120 kg) were randomly divided into 2 groups, with 13 in each group. 1000 IU shCG (test reagent) or 1000 IU uhCG (reference reagent) was injected into the neck muscle of the shCG group and the uhCG group, and the serum was separated and the blood drug concentration was tested.


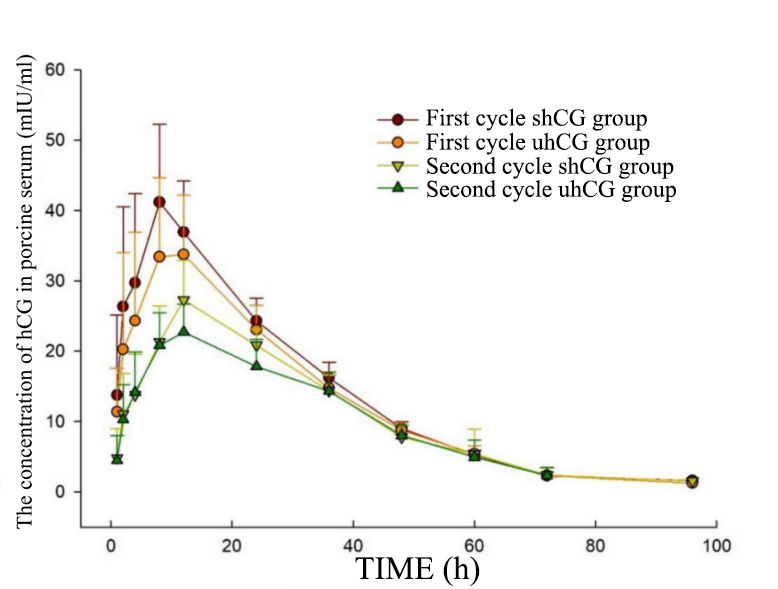


Figure 1: Time-Concentration Curve of the Drug

Appendix F: Pharmacokinetic data for spFSH

Adult sows were given a single intramuscular injection of 0.25 (IM-L), 0.5 (IM-M) and 1 (IM-H) μg/kg spFSH, and a single intravenous injection of 0.5 μg/kg spFSH (IV). Serum was collected and blood drug concentration was tested.


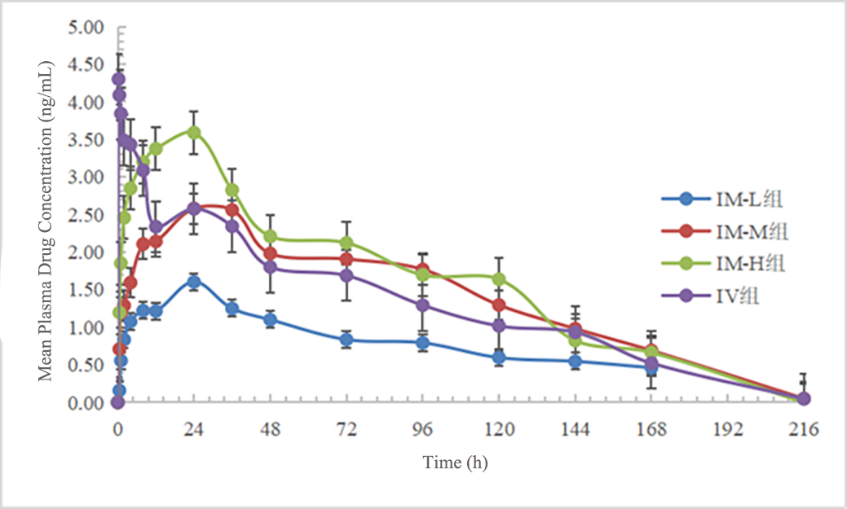
Figure 2 Average blood drug concentrations at different times in the test drug group
